# Risk factors and a prediction model for the prognosis of intracerebral hemorrhage using cerebral microhemorrhage and clinical factors

**DOI:** 10.3389/fneur.2023.1268627

**Published:** 2023-11-23

**Authors:** Hao Feng, Xin Wang, Wenjuan Wang, Xingquan Zhao

**Affiliations:** ^1^Department of Neurology, Beijing Tiantan Hospital, Capital Medical University, Beijing, China; ^2^China National Clinical Research Center for Neurological Diseases, Beijing, China; ^3^Center of Stroke, Beijing Institute for Brain Disorders, Beijing, China; ^4^Research Unit of Artificial Intelligence in Cerebrovascular Disease, Chinese Academy of Medical Sciences, Beijing, China

**Keywords:** risk factors, prediction model, prognosis, intracerebral hemorrhage, cerebral microhemorrhage

## Abstract

**Background:**

This study aimed to identify the risk factors and construct a prediction model for the prognosis of intracerebral hemorrhage (ICH) at discharge, 3 months, and 12 months.

**Methods:**

A total of 269 patients with ICH were retrospectively enrolled at our hospital between January 2014 and August 2016. The prognosis of ICH was assessed using the modified Rankin Scale (mRS); an mRS score > 2 was considered a poor outcome. The primary endpoint was the 3-month mRS, whereas the secondary endpoints included the mRS scores at discharge and 12 months, and mortality.

**Results:**

The Glasgow Coma Scale (GCS), National Institutes of Health (NIH) stroke scale, International Normalized Ratio (INR), blood urea nitrogen (BUN), epencephalon hemorrhage, and primary hematoma volume were significantly associated with a poor mRS score at 3 months. The predictive value of the prediction model based on these factors for a poor mRS score was 87.8%. Furthermore, a poor mRS score at discharge was affected by the GCS, NIH stroke scale, and primary hematoma volume; the constructed model based on these factors had a predictive value of 87.6%. In addition, the GCS, NIH stroke scale, and surgery were significantly related to a poor mRS score at 12 months; the predictive value of the constructed model based on the aforementioned factors for a poor mRS score was 86.5%. Finally, primary hematoma volume is significantly associated with the risk of 12 months mortality.

**Conclusions:**

The study identified risk factors and constructed a prediction model for poor mRS scores and mortality at discharge, 3 and 12 months in patients with ICH. The prediction models for mRS scores showed a relatively high predictive performance.

## 1 Introduction

Stroke accounted for 12.2 million incident case of stroke in 2019, and 101 million prevalent cases of stroke (1). In China, there was an estimated 17.8 million adults presented a stroke, and 2.3 million of cases dying as a result (2–4). Stroke can be classified as ischemic or hemorrhagic, with the latter including intracerebral hemorrhage (ICH) and subarachnoid hemorrhage. ICH accounts for 15–20% of stroke cases, with an annual incidence of 24.3 per 100,000 population, and the disability rate can reach as high as 75% (5, 6). Currently, there are no effective therapeutic strategies for ICH, and prognostic factors, such as hyperglycemia, hypertension, hematoma volume, and hematoma expansion, need to be identified to improve the prognosis of ICH (7).

The prognostic factors for ICH have already been identified, including older age, higher blood plasma glutamate, tumor necrosis factor alpha (TNF-α), initial ICH volume, Scandinavian Stroke Scale score, dialysis, diabetes, Glasgow Coma Scale (GCS) score, bilateral dilated pupils, higher international normalized ratio (INR), hematoma in the cerebellum or brainstem, hematoma volume, and the presence of intraventricular hematoma (8–10). However, these studies did not assess the role of cerebral microhemorrhages (CMs) in ICH prognosis. CMs have been observed in 5% of healthy elderly individuals; the risk factors for CMs include a history of stroke or dementia, hypertension, and diabetes (11). Studies have illustrated that the number and location of CMs are significantly associated with the progression of ICH (12, 13); however, whether they affect the prognosis of ICH remains unclear.

Therefore, we conducted this study to identify the potential prognostic factors for ICH and constructed a predictive model for functional status based on CMs and clinical factors.

## 2 Methods

### 2.1 Study design and population

Patients with ICH admitted to our hospital between January 2014 and August 2016 were retrospectively collected. This study was approved by the institutional review board of Beijing Tiantan Hospital, Capital Medical University (KY2014-023-02), and informed consent was signed from all patients after explaining the purpose of the study. All procedures performed in studies involving human participants were in accordance with the ethical standards of the institutional and/or national research committee and with the 1964 Helsinki declaration and its later amendments or comparable ethical standards. Patients were included if the following criteria were met: (1) adult patients (age ≥ 18.0 years); (2) patients firstly presenting with the onset of ICH; (3) patients admitted in our department within 24 h; (4) a diagnosis of ICH on the basis of brain computerized tomography; and (5) available information regarding CMs, clinical factors, and modified Rankin Scale (mRS) at discharge, 3 and 12 months. Patients were excluded if they (1) had a primary ventricular hemorrhage; (2) were diagnosed with secondary ICH, including head trauma, brain tumor, aneurysm, cavernous hemangioma, arteriovenous malformations, acute thrombolysis, coagulopathy, or moyamoya disease; or (3) had incomplete information regarding CMs or outcomes.

### 2.2 Data collection and variable definition

Patients information was collected from electronic medical records, including age, sex, time from onset to admission, smoking, alcohol, body mass index (BMI), hypertension, diabetes mellitus (DM), hyperlipidemia, history of ischemic stroke, history of hemorrhagic stroke, history of subarachnoid hemorrhage, antiplatelet drugs, anticoagulant drugs, antihypertensive drugs, lipid-lowering drugs, antidiabetic drugs, systolic blood pressure (SBP), diastolic blood pressure (DBP), GCS, National Institutes of Health (NIH) stroke scale, white blood cell (WBC), platelet, fasting glucose, INR, creatinine, blood urea nitrogen (BUN), total cholesterol (TC), triglyceride (TG), high density lipoprotein (HDL), low density lipoprotein (LDL), glytamic-pyruvic transaminase (ALT), glutamic oxalacetic transaminase (AST), alkaline phosphatase (ALP), surgical treatment, location, primary hematoma volume, deep CMs, lobar CMs, subtentorial CMs, and total CMs. Blood samples were obtained in all patients within 1 h after admission. The fasting lipid profiles were obtained on the second day of admission. The susceptibility-weighted imaging showed very low signal intensity lesions was defined as CMs. The signal of CMs was similar to the venous on the susceptibility-weighted imaging phase image, and opposite the calcification signal. The inter- and intra-observer reliability for detecting CMs with Cohen's kappa values of 0.96 and 0.98, respectively. Similarly, the Cohen's kappa values of inter- and intra-observer reliability for assessing the location of CMs were 0.96 and 0.98, respectively. The modified Rankin Scale (mRS) was used to assess the prognosis of ICH, and a poor outcome was defined as an mRS score > 2 (14). Functional outcomes (mRS) were assessed after discharge, at 3 months, and at 12 months. The primary endpoint was the 3-months mRS, whereas the secondary endpoints included the mRS scores at discharge and 12 months, and mortality.

### 2.3 Statistical analysis

The baseline characteristics of the patients with ICH with good or poor functional outcomes were assigned as continuous and categorical variables, respectively. Continuous variables are presented as mean (standard deviation) or median (interquartile range) according to the data distribution, while categorical variables are shown as number and frequency. Differences between good and poor functional outcomes were analyzed using the independent *t* test, Kruskal–Wallis test, or chi-square test. Univariate logistic regression analysis was performed to identify potential risk factors, and the factors were subjected to the multivariate logistic regression analysis using α = 0.05 and β = 0.10. The deep CMs, lobar CMs, subtentorial CMs, and total CMs were mandatory inclusion model, and the prediction model for functional outcomes at discharge, at 3 months, and 12 months was assessed using the receiver operating characteristic (ROC) curve with the area under the curve (AUC). All tests were two-sided, and *P* < 0.05 was regarded as statistically significant. SPSS version 18 for Windows (SPSS, Chicago, IL, USA) was used to perform statistical analyses.

## 3 Results

### 3.1 Baseline characteristics

Of 269 included patients, the mean age was 56.05 years, and 71% of included patients were male. The median time from onset to admitted was 5.16 h. Thirteen patients (4.83%) received surgical treatment, while the remaining patients treated with conservative therapy. The baseline characteristics according to the 3-month functional outcomes are shown in Table 1. There were significant differences between mRS ≤ 2 and mRS > 2 for age (*P* = 0.002), time from onset to admission (*P* = 0.029), the use of antidiabetic drugs (*P* = 0.015), GCS (*P* < 0.001), NIH stroke scale (*P* < 0.001), WBC (*P* = 0.026), fasting glucose (*P* < 0.001), INR (*P* = 0.009), BUN (*P* = 0.041), LDL (*P* = 0.002), and primary hematoma volume (*P* < 0.001). However, we did not find significant differences between the groups regarding sex, smoking, alcohol consumption, BMI, hypertension, DM, hyperlipidemia, history of ischemic stroke, history of hemorrhagic stroke, history of subarachnoid hemorrhage, antiplatelet drugs, anticoagulant drugs, antihypertensive drugs, lipid-lowering drugs, SBP, DBP, platelet, creatinine, TC, TG, HDL, ALT, AST, ALP, surgical treatment, location, deep CMs, lobar CMs, subtentorial CMs, and total CMs.

**Table 1:** The baseline characteristics of collected patients.

**Variable**	**Overall (*n* = 269)**	**3 months mRS**		
		**Good (** ≤ **2) (*****n*** = **175)**	**Poor (**>**2) (*****n*** = **94)**	***P*** **-value**
Age (years)	56.05 (13.09)	54.21 (12.79)	59.47 (13.03)	0.002
**Sex**				0.942
Female	78 (29.00)	51 (29.14)	27 (28.72)	
Male	191 (71.00)	124 (70.86)	67 (71.28)	
Time from onset to admitted (hours)	5.16 (2.52, 15.33)	5.80 (2.65, 16.73)	4.50 (2.18, 7.85)	0.029
**Smoking**				0.990
Never	134 (49.81)	87 (49.71)	47 (50.00)	
Current	94 (34.94)	62 (35.43)	32 (34.04)	
Ever	36 (13.38)	23 (13.14)	13 (13.83)	
Unclear	5 (1.86)	3 (1.71)	2 (2.13)	
**Alcohol**				0.325
Yes	138 (51.30)	94 (53.71)	44 (46.81)	
No	126 (46.84)	79 (45.14)	47 (50.00)	
Unclear	5 (1.86)	2 (1.14)	3 (3.19)	
BMI (kg/m^2^)	25.24 (22.86, 27.68)	25.24 (23.03, 27.76)	25.21 (22.60, 27.44)	0.683
Hypertension	191 (71.27)	124 (71.26)	67 (71.28)	0.998
Diabetes mellitus	38 (14.13)	28 (16.00)	10 (10.64)	0.229
Hyperlipidemia	30 (11.15)	22 (12.57)	8 (8.51)	0.313
History of ischemic stroke	38 (14.13)	22 (12.57)	16 (17.02)	0.318
History of hemorrhagic stroke	6 (2.23)	2 (1.14)	4 (4.26)	0.224
History of subarachnoid hemorrhage	1 (0.37)	1 (0.57)	0 (0.00)	1.000
**Antiplatelet drugs**				0.613
Yes	37 (13.75)	23 (13.14)	14 (14.89)	
No	219 (81.41)	145 (82.86)	74 (78.72)	
Unclear	13 (4.83)	7 (4.00)	6 (6.38)	
**Anticoagulant drugs**				0.100
Yes	0 (0.00)	0 (0.00)	0 (0.00)	
No	263 (97.77)	173 (98.86)	90 (95.74)	
Unclear	6 (2.23)	2 (1.14)	4 (4.26)	
**Antihypertensive drugs**				0.882
Yes	93 (34.57)	61 (34.86)	32 (34.04)	
No	164 (60.97)	107 (61.14)	57 (60.64)	
Unclear	12 (4.46)	7 (4.00)	5 (5.32)	
**Lipid-lowering drugs**				0.204
Yes	21 (7.81)	16 (9.14)	5 (5.32)	
No	235 (87.36)	153 (87.43)	82 (87.23)	
Unclear	13 (4.83)	6 (3.43)	7 (7.45)	
**Antidiabetic drug**				0.015
Yes	22 (8.18)	19 (10.86)	3 (3.19)	
No	238 (88.48)	153 (87.43)	85 (90.43)	
Unclear	9 (3.35)	3 (1.71)	6 (6.38)	
SBP (mmHg)	162.99 (24.81)	161.50 (25.07)	165.79 (24.19)	0.188
DBP (mmHg)	95.00 (80.00, 108.00)	95.00 (80.00, 109.00)	96.00 (80.00, 106.00)	0.903
GCS	15.00 (13.00, 15.00)	15.00 (14.00, 15.00)	14.00 (10.00, 15.00)	< 0.001
NIH stroke scale	7.00 (3.00, 13.00)	4.00 (1.00, 9.00)	13.00 (9.00, 18.00)	< 0.001
WBC (^*^10^9^/L)	9.46 (7.53, 11.67)	9.14 (7.35, 11.24)	10.02 (7.73, 13.11)	0.026
Platelet (^*^10^9^/L)	215.50 (181.00, 257.00)	218.00 (188.00, 254.00)	209.00 (162.00, 260.00)	0.236
Fasting glucose (mmol/L)	5.21 (4.47, 6.04)	4.93 (4.31, 5.80)	5.80 (4.86, 6.44)	< 0.001
INR	0.95 (0.91, 1.00)	0.94 (0.90, 1.00)	0.96 (0.92, 1.02)	0.009
Creatinine (μmoI/L)	61.54 (51.38, 72.40)	63.00 (51.79, 72.40)	60.57 (50.27, 72.25)	0.525
BUN (mmol/L)	5.10 (4.15, 6.20)	5.00 (4.10, 6.00)	5.20 (4.30, 6.50)	0.041
TC (mmol/L)	4.70 (4.07, 5.43)	4.80 (4.09, 5.56)	4.55 (3.95, 5.21)	0.054
TG (mmol/L)	1.33 (1.00, 1.75)	1.42 (1.04, 1.83)	1.21 (0.93, 1.63)	0.060
HDL (mmol/L)	1.15 (1.00, 1.42)	1.15 (0.98, 1.40)	1.19 (1.02, 1.51)	0.103
LDL (mmol/L)	3.05 (2.43, 3.63)	3.16 (2.54, 3.77)	2.86 (2.15, 3.33)	0.002
ALT (U/L)	27.05 (20.00, 34.20)	26.70 (19.60, 34.10)	27.80 (21.70, 34.20)	0.511
AST (U/L)	21.35 (17.90, 26.90)	21.10 (17.90, 25.10)	22.40 (17.90, 28.60)	0.107
ALP (U/L)	82.90 (65.40, 98.20)	83.00 (64.30, 97.10)	81.75 (65.90, 105.30)	0.778
**Surgical treatment**				0.078
No	256 (95.17)	170 (97.14)	86 (91.49)	
Yes	13 (4.83)	5 (2.86)	8 (8.51)	
**Location**				0.194
Lobar	80 (30.89)	60 (35.50)	20 (22.22)	
Basal ganglia	111 (42.86)	66 (39.05)	45 (50.00)	
Thalamus	44 (16.99)	26 (15.38)	18 (20.00)	
Brainstem	13 (5.02)	9 (5.33)	4 (4.44)	
Epencephalon	11 (4.25)	8 (4.73)	3 (3.33)	
Primary hematoma volume	12.50 (4.90, 28.10)	9.60 (3.80, 26.50)	20.43 (8.90, 40.10)	< 0.001
**Deep CMs**				0.134
No	161 (59.85)	99 (56.57)	62 (65.96)	
Yes	108 (40.15)	76 (43.43)	32 (34.04)	
**Lobar CMs**				0.940
No	221 (82.16)	144 (82.29)	77 (81.91)	
Yes	48 (17.84)	31 (17.71)	17 (18.09)	
**Subtentorial CMs**				0.705
No	217 (80.67)	140 (80.00)	77 (81.91)	
Yes	52 (19.33)	35 (20.00)	17 (18.09)	
**Total CMs**				0.519
No	133 (49.44)	84 (48.00)	49 (52.13)	
Yes	136 (50.56)	91 (52.00)	45 (47.87)	

ALP, alkaline phosphatase; ALT, glytamic-pyruvic transaminase; AST, glutamic oxalacetic transaminase; BMI, body mass index; BUN, blood urea nitrogen; CMs, cerebral microhemorrhages; DBP, diastolic blood pressure; GCS, Glasgow Coma Scale; HDL, high density lipoprotein; INR, international normalized ratio; LDL, low density lipoprotein; mRS, Modified Rankin Scale; NIH, National Institutes of Health; SBP, systolic blood pressure; TC, total cholesterol; TG, triglyceride; WBC, white blood cell.

### 3.2 Three-month mRS

**Figure 1:**
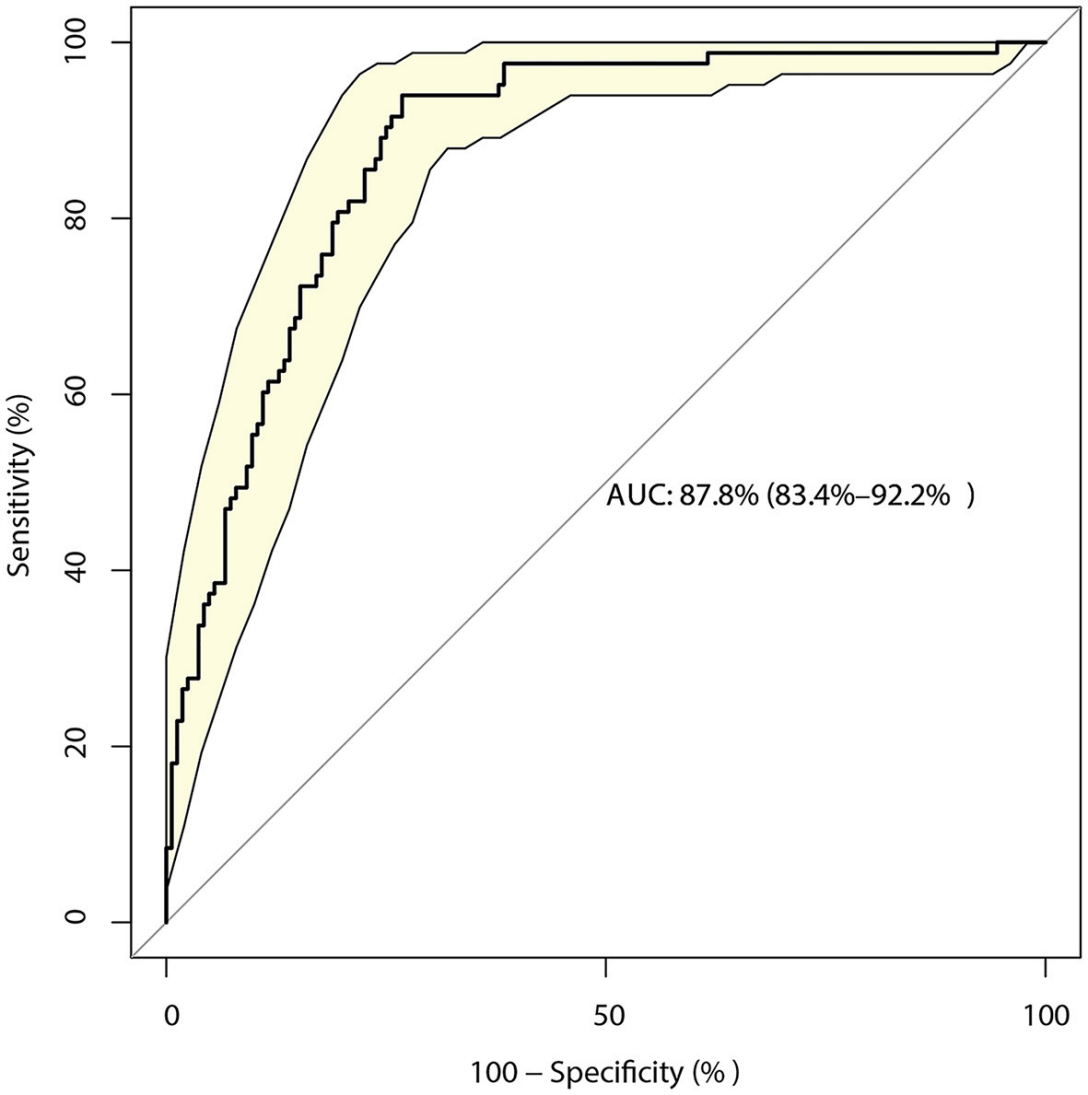
Receiver operating characteristic curve for the risk of a 3-month modified Rankin Scale (mRS) score > 2 in patients with intracerebral hemorrhage (ICH), including the six-component risk factor model.

The identified risk factors for the 3-month mRS are shown in Table 2. The univariate analysis found that poor functional outcomes could be affected by age [odds ratio (OR): 1.03; *P* = 0.002], time from onset to admission (OR: 0.97; *P* = 0.011), no use of antidiabetic drugs (*P* = 0.048) or unclear use of antidiabetic drugs (*P* = 0.007), GCS (*P* < 0.001), NIH stroke scale (*P* < 0.001), WBC (*P* = 0.005), INR (*P* = 0.023), BUN (*P* = 0.046), LDL (*P* = 0.005), AST (*P* = 0.031), surgical treatment (*P* = 0.049), basal ganglia hemorrhage (*P* = 0.027), and primary hematoma volume (*P* < 0.001). After adjusting for potential confounders, we noted that the GCS (OR: 1.93; 95% CI: 1.33–2.79; *P* < 0.001), NIH stroke scale (OR: 1.53; 95% CI: 1.32–1.78; *P* < 0.001), INR (OR: 1804.29; 95% CI: 6.44–505855.5; *P* = 0.009), BUN (OR: 1.47; 95% CI: 1.08–2.01; *P* = 0.014), epencephalon hemorrhage (OR: 115.80; 95% CI: 9.89–1356.22; *P* < 0.001), and primary hematoma volume (OR: 1.05; 95% CI: 1.01–1.08; *P* = 0.007) were significantly associated with a poor 3-month mRS score. The predictive value of the prediction model based on these factors was 87.8% (95% CI: 83.4–92.2%; Figure 1).

**Table 2:** The risk factors for 3-months mRS in ICH patients.

**Variable**	**Univariate logistic analysis**			**Multivariate logistic analysis**		
	β **value**	**OR (95%CI)**	***P*** **-value**	β **value**	**OR (95%CI)**	***P*** **-value**
Age	0.032	1.03 (1.01–1.05)	0.002			
Sex	−0.020	0.98 (0.56–1.70)	0.942			
Time from onset to admitted	−0.029	0.97 (0.95–0.99)	0.011			
**Smoking**						
Never	Ref	-	-			
Current	−0.046	0.96 (0.55–1.66)	0.872			
Ever	0.045	1.05 (0.49–2.25)	0.908			
Unclear	0.210	1.23 (0.20–7.65)	0.821			
**Alcohol**						
Yes	Ref	-	-			
No	0.240	1.27 (0.76–2.11)	0.355			
Unclear	1.164	3.20 (0.52–19.86)	0.211			
BMI (kg/m^2^)	−0.015	0.99 (0.92–1.05)	0.658			
Hypertension	0.001	1.00 (0.57–1.74)	0.998			
Diabetes mellitus	−0.470	0.63 (0.29–1.35)	0.232			
Hyperlipidemia	−0.435	0.65 (0.28–1.52)	0.316			
History of ischemic stroke	0.355	1.43 (0.71–2.87)	0.319			
History of hemorrhagic stroke	1.347	3.84 (0.69–21.39)	0.124			
History of subarachnoid hemorrhage	−12.717	0.00 (0.00)	0.987			
**Antiplatelet drugs**						
Yes	Ref	-	-			
No	−0.176	0.84 (0.41–1.72)	0.632			
Unclear	0.342	1.41 (0.39–5.05)	0.599			
Anticoagulant drugs (unclear vs. no)	1.347	3.84 (0.69–21.39)	0.124			
**Antihypertensive drugs**						
Yes	Ref	-	-			
No	0.015	1.02 (0.59–1.73)	0.955			
Unclear	0.309	1.36 (0.40–4.63)	0.621			
**Lipid-lowering drugs**						
Yes	Ref	-	-			
No	0.539	1.72 (0.61–4.85)	0.309			
Unclear	1.317	3.73 (0.85–16.44)	0.082			
**Antidiabetic drug**						
Yes	Ref	-	-			
No	1.258	3.52 (1.01–12.23)	0.048			
Unclear	2.539	12.66 (2.00–80.12)	0.007			
SBP (mmHg)	0.007	1.01 (1.00–1.02)	0.188			
DBP (mmHg)	−0.002	1.00 (0.98–1.01)	0.815			
GCS	−0.241	0.79 (0.71–0.87)	< 0.001	0.656	1.93 (1.33–2.79)	< 0.001
NIH stroke scale	0.175	1.19 (1.14–1.25)	< 0.001	0.428	1.53 (1.32–1.78)	< 0.001
WBC (^*^10^9^/L)	0.107	1.11 (1.03–1.20)	0.005			
Platelet (^*^10^9^/L)	−0.002	1.00 (0.99–1.00)	0.332			
Fasting glucose (mmol/L)	0.077	1.08 (0.96–1.22)	0.204			
INR	3.580	35.87 (1.65–779.98)	0.023	7.498	1804.29 (6.44–505855.5)	0.009
Creatinine (μmoI/L)	−0.005	0.99 (0.98–1.01)	0.405			
BUN (mmol/L)	0.155	1.17 (1.00–1.36)	0.046	0.387	1.47 (1.08–2.01)	0.014
TC (mmol/L)	−0.224	0.80 (0.61–1.05)	0.108			
TG (mmol/L)	−0.372	0.69 (0.44–1.08)	0.107			
HDL (mmol/L)	0.454	1.57 (0.89–2.79)	0.121			
LDL (mmol/L)	−0.468	0.63 (0.45–0.87)	0.005			
ALT (U/L)	0.003	1.00 (0.99–1.01)	0.563			
AST (U/L)	0.024	1.02 (1.00–1.05)	0.031			
ALP (U/L)	0.001	1.00 (0.99–1.01)	0.716			
Surgical treatment	−1.151	0.32 (0.10–1.00)	0.049			
**Location**						
Lobar	Ref	-	-			
Basal ganglia	0.716	2.05 (1.09–3.85)	0.027	1.045	2.84 (0.73–11.13)	0.134
Thalamus	0.731	2.08 (0.95–4.56)	0.068	1.678	5.35 (0.94–30.42)	0.058
Brainstem	0.288	1.33 (0.37–4.80)	0.660	1.129	3.09 (0.10–92.56)	0.515
Epencephalon	0.118	1.12 (0.27–4.65)	0.871	4.752	115.80 (9.89–1356.22)	< 0.001
Primary hematoma volume	0.021	1.02 (1.01–1.03)	< 0.001	0.045	1.05 (1.01–1.08)	0.007
Deep CMs	−0.397	0.67 (0.40–1.13)	0.135	−0.391	0.68 (0.12–3.82)	0.658
Lobar CMs	0.025	1.03 (0.53–1.97)	0.939	1.292	3.64 (0.83–15.98)	0.087
Subtentorial CMs	−0.124	0.88 (0.46–1.68)	0.705	1.217	3.38 (0.74–15.32)	0.115
Total CMs	−0.165	0.85 (0.51–1.40)	0.519	−1.416	0.24 (0.03–1.83)	0.170

### 3.3 mRS at discharge

**Figure 2:**
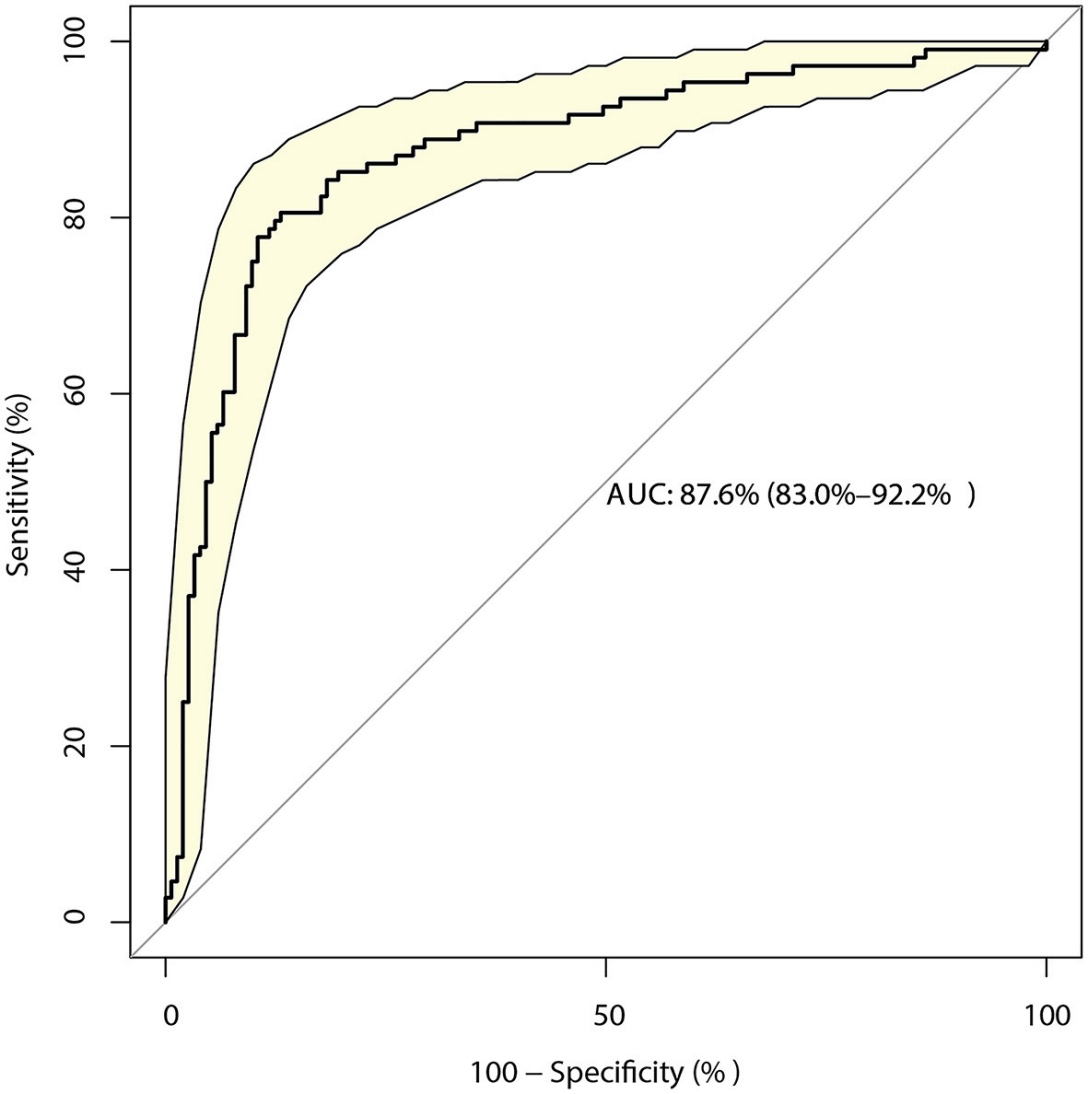
Receiver operating characteristic curve for the risk of a modified Rankin Scale (mRS) score > 2 at discharge in patients with intracerebral hemorrhage (ICH), including the three-component risk factor model.

The identified risk factors for the mRS score at discharge are presented in Table 3. The univariate analyses revealed that the time from onset to admission (*P* = 0.018), GCS score (*P* < 0.001), NIH stroke scale score (*P* < 0.001), AST level (*P* = 0.002), surgical treatment (*P* = 0.016), basal ganglia hemorrhage (*P* = 0.022), and primary hematoma volume (*P* < 0.001) were significantly associated with poor functional outcomes. After adjusting for potential confounders, the mRS at discharge could be affected by the GCS (OR: 1.48; 95% CI: 1.08–2.03; *P* = 0.015), NIH stroke scale (OR: 1.47; 95% CI: 1.30–1.66; *P* < 0.001), and primary hematoma volume (OR: 1.04; 95% CI: 1.01–1.06; *P* = 0.002). The model constructed based on these factors had a predictive value of 87.6% (95% CI: 83.0–92.2%; Figure 2).

**Table 3:** The risk factors for mRS at discharge in ICH patients.

**Variable**	**Univariate logistic analysis**			**Multivariate logistic analysis**		
	β **value**	**OR (95%CI)**	***P*** **-value**	β **value**	**OR (95%CI)**	***P*** **-value**
Age	0.012	1.01 (0.99–1.03)	0.221			
Sex	−0.110	0.90 (0.52–1.53)	0.688			
Time from onset to admitted	−0.024	0.98 (0.96–1.00)	0.018			
**Smoking**						
Never	Ref	-	-			
Current	0.382	1.46 (0.86–2.49)	0.160			
Ever	−0.269	0.76 (0.35–1.66)	0.496			
Unclear	−0.962	0.38 (0.04–3.51)	0.395			
**Alcohol**						
Yes	Ref	-	-			
No	0.219	1.24 (0.76–2.03)	0.382			
Unclear	0.036	1.04 (0.17–6.41)	0.969			
BMI (kg/m^2^)	−0.023	0.98 (0.92–1.04)	0.487			
Hypertension	0.164	1.18 (0.69–2.02)	0.551			
Diabetes mellitus	−0.104	0.90 (0.45–1.82)	0.771			
Hyperlipidemia	−0.569	0.57 (0.25–1.29)	0.175			
History of ischemic stroke	−0.104	0.90 (0.45–1.82)	0.771			
History of hemorrhagic stroke	0.346	1.41 (0.28–7.13)	0.676			
History of subarachnoid hemorrhage	−12.913	0.00 (0.00)	0.986			
**Antiplatelet drugs**						
Yes	Ref	-	-			
No	0.042	1.04 (0.51–2.12)	0.908			
Unclear	0.229	1.26 (0.35–4.49)	0.725			
Anticoagulant drugs (unclear vs. no)	1.986	7.29 (0.84–63.27)	0.072			
**Antihypertensive drugs**						
Yes	Ref	-	-			
No	0.120	1.13 (0.67–1.89)	0.651			
Unclear	0.078	1.08 (0.32–3.66)	0.900			
**Lipid-lowering drugs**						
Yes	Ref	-	-			
No	0.598	1.82 (0.68–4.85)	0.232			
Unclear	1.070	2.92 (0.69–12.36)	0.146			
**Antidiabetic drug**						
Yes	Ref	-	-			
No	0.011	1.01 (0.42–2.46)	0.981			
Unclear	0.591	1.81 (0.38–8.64)	0.459			
SBP (mmHg)	0.002	1.00 (0.99–1.01)	0.681			
DBP (mmHg)	−0.006	0.99 (0.98–1.01)	0.408			
GCS	−0.335	0.72 (0.63–0.81)	< 0.001	0.393	1.48 (1.08–2.03)	0.015
NIH stroke scale	0.234	1.26 (1.19–1.34)	< 0.001	0.384	1.47 (1.30–1.66)	< 0.001
WBC (^*^10^9^/L)	0.058	1.06 (0.99–1.14)	0.111			
Platelet (^*^10^9^/L)	−0.001	1.00 (1.00–1.00)	0.756			
Fasting glucose (mmol/L)	0.085	1.09 (0.97–1.23)	0.163			
INR	0.813	2.25 (0.14–36.05)	0.565			
Creatinine (μmoI/L)	−0.007	0.99 (0.98–1.01)	0.273			
BUN (mmol/L)	0.024	1.02 (0.89–1.18)	0.745			
TC (mmol/L)	0.025	1.03 (0.80–1.32)	0.844			
TG (mmol/L)	−0.393	0.68 (0.44–1.04)	0.074			
HDL (mmol/L)	0.235	1.26 (0.84–1.90)	0.259			
LDL (mmol/L)	−0.062	0.94 (0.71–1.25)	0.674			
ALT (U/L)	0.010	1.01 (1.00–1.02)	0.118			
AST (U/L)	0.042	1.04 (1.02–1.07)	0.002			
ALP (U/L)	0.001	1.00 (0.99–1.01)	0.824			
Surgical treatment	−1.616	0.20 (0.05–0.74)	0.016			
**Location**						
Lobar	Ref	-	-			
Basal ganglia	0.692	2.00 (1.10–3.62)	0.022			
Thalamus	0.492	1.64 (0.77–3.47)	0.200			
Brainstem	0.204	1.23 (0.37–4.11)	0.740			
Epencephalon	−13.569	0.00 (0.00)	0.971			
Primary hematoma volume	0.033	1.03 (1.02–1.05)	< 0.001	0.036	1.04 (1.01–1.06)	0.002
Deep CMs	−0.126	0.88 (0.54–1.45)	0.620	0.105	1.11 (0.20–6.22)	0.905
Lobar CMs	−0.429	0.65 (0.34–1.26)	0.200	−0.049	0.95 (0.20–4.53)	0.951
Subtentorial CMs	−0.472	0.62 (0.33–1.18)	0.147	0.276	1.32 (0.30–5.72)	0.713
Total CMs	−0.283	0.75 (0.46–1.22)	0.253	−1.163	0.31 (0.04–2.25)	0.248

### 3.4 Twelve-month mRS

**Figure 3:**
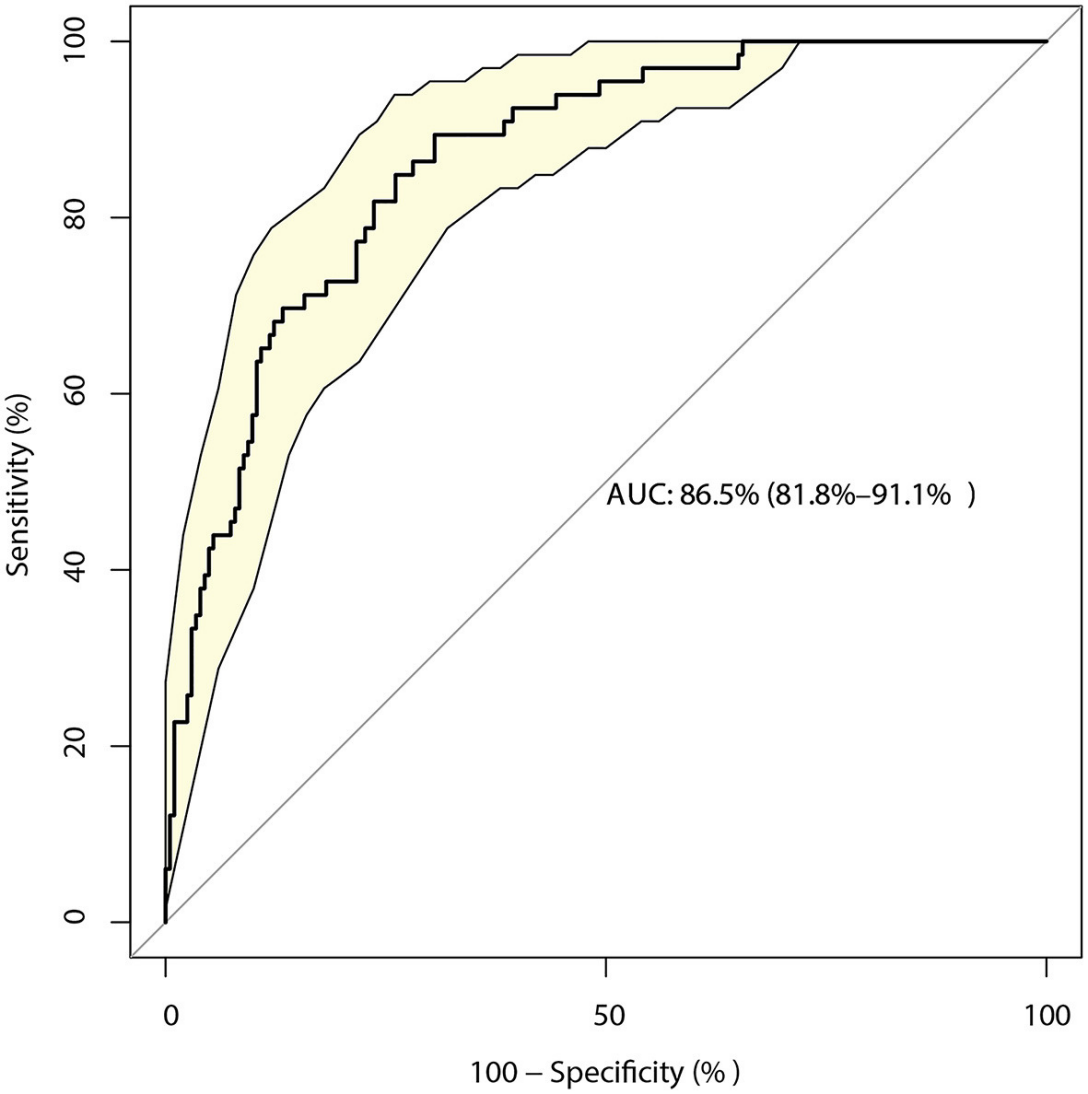
Receiver operating characteristic curve for the risk of a 12-month modified Rankin Scale (mRS) score > 2 in patients with intracerebral hemorrhage (ICH), including the three-component risk factor model.

The identified risk factors for the mRS score at 12 months are presented in Table 4. The univariate analyses found that the 12-month mRS score was affected by age (*P* < 0.001), history of ischemic stroke (*P* = 0.008), unclear information regarding the use of antidiabetic drugs (*P* = 0.012), GCS (*P* < 0.001), NIH stroke scale (*P* < 0.001), TC (*P* = 0.006), LDL (*P* = 0.002), surgical treatment (*P* = 0.004), and primary hematoma volume (*P* < 0.001). After adjusting for potential confounders, we noted that the GCS (OR: 2.18; 95% CI: 1.41–3.38; *P* < 0.001), NIH stroke scale (OR: 1.54; 95% CI: 1.30–1.81; *P* < 0.001), and treated with surgery (OR: 0.04; 95% CI: 0.00–0.29; *P* = 0.002) were significantly associated with a poor mRS after 12 months. The prediction model based on the above factors had a predictive value of 86.5% (95% CI: 81.8–91.1%; Figure 3).

**Table 4:** The risk factors for 12-months mRS in ICH patients.

**Variable**	**Univariate logistic analysis**			**Multivariate logistic analysis**		
	β **value**	**OR (95%CI)**	***P*** **-value**	β **value**	**OR (95%CI)**	***P*** **-value**
Age	0.055	1.06 (1.03–1.08)	< 0.001	0.103	1.11 (1.05–1.17)	< 0.001
Sex	−0.537	0.58 (0.30–1.13)	0.111			
Time from onset to admitted	−0.022	0.98 (0.95–1.00)	0.073			
**Smoking**						
Never	Ref	-	-			
Current	0.370	1.45 (0.78–2.68)	0.238			
Ever	0.376	1.46 (0.63–3.37)	0.381			
Unclear	0.926	2.52 (0.40–15.84)	0.323			
**Alcohol**						
Yes	Ref	-	-			
No	−0.173	0.84 (0.48–1.48)	0.550			
Unclear	1.485	4.41 (0.71–27.51)	0.112			
BMI (kg/m^2^)	−0.052	0.95 (0.88–1.03)	0.184			
Hypertension	0.409	1.51 (0.79–2.88)	0.216			
Diabetes mellitus	−0.229	0.80 (0.35–1.83)	0.591			
Hyperlipidemia	−0.293	0.75 (0.29–1.91)	0.542			
History of ischemic stroke	0.968	2.63 (1.29–5.39)	0.008			
History of hemorrhagic stroke	1.155	3.17 (0.63–16.12)	0.164			
History of subarachnoid hemorrhage	−12.462	0.00 (0.00)	0.989			
**Antiplatelet drugs**						
Yes	Ref	-	-			
No	−0.358	0.70 (0.32–1.51)	0.364			
Unclear	0.390	1.48 (0.39–5.54)	0.563			
Anticoagulant drugs (unclear vs. no)	1.155	3.17 (0.63–16.12)	0.164			
**Antihypertensive drugs**						
Yes	Ref	-	-			
No	−0.233	0.79 (0.44–1.42)	0.437			
Unclear	0.308	1.36 (0.38–4.92)	0.639			
**Lipid-lowering drugs**						
Yes	Ref	-	-			
No	−0.022	0.98 (0.34–2.79)	0.966			
Unclear	1.009	2.74 (0.62–12.08)	0.182			
**Antidiabetic drug**						
Yes	Ref	-	-			
No	1.192	3.29 (0.75–14.51)	0.115			
Unclear	2.525	12.49 (1.76–88.65)	0.012			
SBP (mmHg)	0.006	1.01 (0.99–1.02)	0.280			
DBP (mmHg)	−0.007	0.99 (0.98–1.01)	0.387			
GCS	−0.237	0.79 (0.71–0.87)	< 0.001	0.781	2.18 (1.41–3.38)	< 0.001
NIH stroke scale	0.143	1.15 (1.10–1.21)	< 0.001	0.431	1.54 (1.30–1.81)	< 0.001
WBC (^*^10^9^/L)	0.008	1.01 (0.93–1.09)	0.842			
Platelet (^*^10^9^/L)	−0.005	1.00 (0.99–1.00)	0.073			
Fasting glucose (mmol/L)	0.122	1.13 (1.00–1.28)	0.055			
INR	1.915	6.78 (0.25–185.68)	0.257			
Creatinine (μmoI/L)	−0.001	1.00 (0.99–1.01)	0.860			
BUN (mmol/L)	0.137	1.15 (0.98–1.35)	0.095			
TC (mmol/L)	−0.481	0.62 (0.44–0.87)	0.006			
TG (mmol/L)	−0.430	0.65 (0.38–1.11)	0.116			
HDL (mmol/L)	0.177	1.19 (0.86–1.66)	0.290			
LDL (mmol/L)	−0.621	0.54 (0.36–0.80)	0.002			
ALT (U/L)	−0.001	1.00 (0.99–1.01)	0.923			
AST (U/L)	0.017	1.02 (1.00–1.04)	0.115			
ALP (U/L)	0.005	1.00 (1.00–1.01)	0.219			
Surgical treatment	−1.698	0.18 (0.06–0.58)	0.004	−3.321	0.04 (0.00–0.29)	0.002
**Location**						
Lobar	Ref	-	-			
Basal ganglia	0.300	1.35 (0.67–2.70)	0.398			
Thalamus	0.517	1.68 (0.72–3.92)	0.232			
Brainstem	0.575	1.78 (0.49–6.52)	0.385			
Epencephalon	−0.916	0.40 (0.05–3.36)	0.399			
Primary hematoma volume	0.025	1.03 (1.01–1.04)	< 0.001			
Deep CMs	−0.042	0.96 (0.54–1.69)	0.886	−1.516	0.22 (0.04–1.37)	0.104
Lobar CMs	0.655	1.92 (0.98–3.77)	0.056	1.152	3.16 (0.62–16.10)	0.165
Subtentorial CMs	0.031	1.03 (0.51–2.08)	0.931	0.360	1.43 (0.31–6.71)	0.647
Total CMs	0.293	1.34 (0.77–2.34)	0.304	0.842	2.32 (0.29–18.37)	0.425

### 3.5 Mortality

The number of death occurred at discharge, 3 and 12-months were 1, 5, and 13, respectively. Considering lower incidences of mortality at discharge, and 3-months, the risk factors for 12-months were identified and shown in Table 5. The univariate analyses found that the 12-month mortality was affected by age (*P* = 0.002), history of ischemic stroke (*P* < 0.001), GCS (*P* = 0.046), platelet (*P* = 0.011), LDL (*P* = 0.049), and primary hematoma volume (*P* = 0.024). After adjusting for potential confounders, we noted primary hematoma volume was associated with an increased risk of 12-months mortality (OR: 1.04; 95% CI: 1.00–1.08; *P* = 0.029).

**Table 5:** The risk factors for 12-months mortality in ICH patients.

**Variable**	**Univariate logistic analysis**			**Multivariate logistic analysis**		
	β **value**	**OR (95%CI)**	***P*** **-value**	β **value**	**OR (95%CI)**	***P*** **-value**
Age	0.083	1.09 (1.03–1.14)	0.002	0.046	1.05 (0.94–1.17)	0.405
Sex	−1.641	0.19 (0.03–1.52)	0.118			
Time from onset to admitted	−0.006	0.99 (0.96–1.03)	0.750			
**Smoking**						
Never	Ref	-	-			
Current	−2.095	0.12 (0.01–1.37)	0.088			
Ever	−1.493	0.23 (0.02–2.40)	0.217			
Unclear	−1.012	0.36 (0.03–4.39)	0.426			
**Alcohol**						
Yes	Ref	-	-			
No	−1.402	0.25 (0.03–2.47)	0.233			
Unclear	−2.031	0.13 (0.01–1.46)	0.098			
BMI (kg/m^2^)	−0.016	0.98 (0.90–1.08)	0.728			
Hypertension	0.103	1.11 (0.33–3.71)	0.868			
Diabetes mellitus	−0.707	0.49 (0.06–3.91)	0.503			
Hyperlipidemia	−0.427	0.65 (0.08–5.20)	0.687			
History of ischemic stroke	2.136	8.47 (2.67–26.83)	< 0.001	1.677	5.35 (0.57–49.79)	0.141
History of hemorrhagic stroke	-	-	0.999			
History of subarachnoid hemorrhage	-	-	1.000			
**Antiplatelet drugs**						
Yes	Ref	-	-			
No	0.843	2.32 (0.25–21.37)	0.457			
Unclear	−1.085	0.34 (0.04–3.04)	0.333			
Anticoagulant drugs (unclear vs. no)	1.431	4.18 (0.45–38.66)	0.207			
**Antihypertensive drugs**						
Yes	Ref	-	-			
No	−0.704	0.49 (0.05–4.83)	0.545			
Unclear	−0.573	0.56 (0.07–4.93)	0.605			
**Lipid-lowering drugs**						
Yes	Ref	-	-			
No	0.234	1.26 (0.10–15.50)	0.855			
Unclear	−0.629	0.53 (0.06–4.52)	0.564			
**Antidiabetic drug**						
Yes	Ref	-	-			
No	-	-	0.998			
Unclear	−0.856	0.43 (0.05–3.68)	0.437			
SBP (mmHg)	−0.011	0.99 (0.97–1.01)	0.360			
DBP (mmHg)	−0.026	0.98 (0.94–1.01)	0.167			
GCS	−0.168	0.85 (0.72–1.00)	0.046	0.539	1.72 (0.72–4.07)	0.221
NIH stroke scale	0.054	1.06 (0.99–1.12)	0.092	0.144	1.15 (0.89–1.49)	0.271
WBC (^*^10^9^/L)	−0.137	0.87 (0.71–1.06)	0.177			
Platelet (^*^10^9^/L)	−0.014	0.99 (0.98–1.00)	0.011	−0.009	0.99 (0.97–1.01)	0.350
Fasting glucose (mmol/L)	−0.275	0.76 (0.52–1.12)	0.165			
INR	−0.081	0.92 (0.23–3.78)	0.911			
Creatinine (μmoI/L)	0.005	1.01 (0.98–1.03)	0.651			
BUN (mmol/L)	0.098	1.10 (0.81–1.50)	0.526			
TC (mmol/L)	−0.588	0.56 (0.26–1.21)	0.137			
TG (mmol/L)	−0.325	0.72 (0.23–2.30)	0.582			
HDL (mmol/L)	−0.077	0.93 (0.32–2.65)	0.886			
LDL (mmol/L)	−0.949	0.39 (0.15–1.00)	0.049	−0.916	0.40 (0.10–1.55)	0.185
ALT (U/L)	−0.011	0.99 (0.95–1.03)	0.581			
AST (U/L)	0.007	1.01 (0.97–1.05)	0.741			
ALP (U/L)	−0.016	0.98 (0.96–1.01)	0.255			
Surgical treatment	−0.527	0.59 (0.07–4.92)	0.626			
**Location**						
Lobar	Ref	-	-			
Basal ganglia	-	-	0.999			
Thalamus	-	-	0.999			
Brainstem	-	-	0.999			
Epencephalon	-	-	0.999			
Primary hematoma volume	0.022	1.02 (1.00–1.04)	0.024	0.042	1.04 (1.00–1.08)	0.029
Deep CMs	−1.358	0.26 (0.06–1.19)	0.081	-	-	0.996
Lobar CMs	−0.186	0.83 (0.18–3.87)	0.813	-	-	0.997
Subtentorial CMs	0.237	1.27 (0.34–4.78)	0.726	-	-	0.996
Total CMs	−0.517	0.60 (0.19–1.87)	0.376	-	-	0.997

## 4 Discussion

This study first identified the risk factors for functional outcomes and mortality at discharge, at 3 months, and at 12-months in patients with ICH. A multifactorial predictive model for the risk of poor functional outcomes was constructed, which could be used to screen high-risk patients, and effective treatments could be applied to improve the prognosis of ICH patients. Our study recruited 269 patients with ICH, of which 94 patients reported a 3-month mRS > 2. The 3-month mRS score was affected by the GCS, NIH stroke scale, INR, BUN, epencephalon hemorrhage, and primary hematoma volume. Moreover, the GCS, NIH stroke scale, and primary hematoma volume could affect the mRS at discharge, while the mRS at 12 months could be affected by the GCS, NIH stroke scale, and surgical treatment. Furthermore, the risk of 12-months mortality could affected by primary hematoma volume. The constructed prediction model for mRS scores at discharge, at 3 months, and at 12 months showed a relatively high predictive performance.

Several studies have constructed prediction models for ICH patients (15–19). Fukuda et al. identified 187 patients with aneurysmal subarachnoid hemorrhage and found that a constructed model containing D-dimer was associated with a better discrimination ability for poor outcomes (15). Wang et al. (16) applied an automated machine learning-based approach to construct a prognostic model for patients with ICH and pointed out that the random forest provides the best predictive performance. Katsuki et al. (17) used data from 140 patients with hypertensive ICH and found that the prediction model constructed using a deep learning framework was superior to the model derived from the ICH score, ICH Grading Scale, and FUNC score. Trevisi et al. (18) found that the important features for functional outcomes included the GCS, Charlson Comorbidity Index, ICH score, ICH volume, pupillary status, brainstem location, age, anticoagulant/antiplatelet agents, intraventricular hemorrhage, and cerebellar location, and the discriminative ability was high. Wu et al. (19) identified 83 patients with ICH and found that the combined model applied radiomic scores obtained from intraparenchymal hemorrhage, intraventricular hemorrhage, and clinical characteristics with high accuracy in predicting poor functional outcomes. However, these studies did not systematically identify the risk factors for poor functional outcomes in patients with ICH, and the characteristics of CMs have not been addressed. Thus, the current study was performed to identify risk factors and construct a prediction model for functional outcomes at discharge, at 3 months, and at 12-months in patients with ICH.

Our study found that the risk factors at various time points differed; similar risk factors included the GCS and NIH stroke scale. A potential reason for this could be that these two scales reflect the neurological status of patients with ICH (20, 21). Additional factors potentially influencing functional outcomes included INR, BUN, epencephalon hemorrhage, surgery, and primary hematoma volume. Several reasons could explain these results: (1) elevated INR has already been demonstrated to be associated with a poor prognosis in patients with ICH, especially in patients treated with warfarin (22); (2) BUN is an important index to assess renal function, which could reflect the severity of disease status in patients with ICH (23); (3) hemorrhage location is significantly related to the prognosis of ICH because the invasion sites are associated with the severity of disease (24); and (4) patients treated with surgery are significantly related to hematoma volume, which could affect the prognosis of ICH (25, 26). Therefore, early intervention strategies should be implemented for identified modifiable risk factors in order to improve patient prognosis.

Our study reported the risk of 12-months mortality could affected by primary hematoma volume, which could explained by the primary hematoma volume are significantly related to the severity of disease and subsequent treatments. Moreover, considering the incidences of mortality at discharge and 3-months were lower than expected, thus the power was not enough to detect potential associations. In addition, although our study found that functional outcomes were not affected by CMs, especially deep, lobar, subtentorial, and total CMs, the prediction model based on CMs and clinical factors for mRS > 2 at discharge, 3 and 12 months had a relatively high predictive performance. Considering that the risk factors for poor functional outcomes have already been identified, effective strategies should be applied to patients at a high risk of poor functional outcomes.

This study has some limitations. First, the analysis was based on retrospective data, and the results of our study could have been affected by selection and recall biases. Second, our study was restricted by single-center study with a small sample size, thus the conclusions of this study should be recommended cautiously. Third, the severity of ICH was not restricted as an inclusion criterion, and the prognosis of ICH could be affected by the presence of more severe or mild symptoms. Fourth, the background therapies for ICH differed among the included patients, which could have affected the prognosis of ICH. Fifth, biomarkers levels change over the course of follow-up was not addressed, which needed further explored. Sixth, the predictive model was not verified using an external cohort.

This study identified the risk factors for functional outcomes and mortality at discharge, at 3 months, and at 12 months in patients with ICH, and the CMs were addressed. Moreover, a prediction model was constructed based on the identified risk factors with relatively high predictive performance, which could be applied in clinical practice to identify high-risk patients. Further large-scale prospective studies are required to validate the constructed model.

## Data availability statement

The original contributions presented in the study are included in the article/supplementary material, further inquiries can be directed to the corresponding author.

## Ethics statement

The studies involving humans were approved by the Institutional Review Board of Beijing Tiantan Hospital, Capital Medical University (KY2014-023-02). The studies were conducted in accordance with the local legislation and institutional requirements. The participants provided their written informed consent to participate in this study.

## Author contributions

HF: Conceptualization, Data curation, Formal analysis, Writing—original draft. XW: Data curation, Formal analysis, Writing—review & editing. WW: Data curation, Formal analysis, Writing—review & editing. XZ: Conceptualization, Formal analysis, Funding acquisition, Project administration, Writing—review & editing.
